# Improving the efficiency of plant root system phenotyping through digitization and automation

**DOI:** 10.1270/jsbbs.21053

**Published:** 2022-02-09

**Authors:** Shota Teramoto, Yusaku Uga

**Affiliations:** 1 Institute of Crop Science, National Agriculture and Food Research Organization, Tsukuba, Ibaraki 305-8518, Japan

**Keywords:** root traits, high-throughput, image analysis, semantic segmentation, vectorization

## Abstract

Root system architecture (RSA) determines unevenly distributed water and nutrient availability in soil. Genetic improvement of RSA, therefore, is related to crop production. However, RSA phenotyping has been carried out less frequently than above-ground phenotyping because measuring roots in the soil is difficult and labor intensive. Recent advancements have led to the digitalization of plant measurements; this digital phenotyping has been widely used for measurements of both above-ground and RSA traits. Digital phenotyping for RSA is slower and more difficult than for above-ground traits because the roots are hidden underground. In this review, we summarized recent trends in digital phenotyping for RSA traits. We classified the sample types into three categories: soil block containing roots, section of soil block, and root sample. Examples of the use of digital phenotyping are presented for each category. We also discussed room for improvement in digital phenotyping in each category.

## Introduction

Plants cannot move themselves; therefore, they must efficiently absorb the limited amount of water and nutrients heterogeneously distributed in soil. Efficiently reaching and extracting water and nutrients from the soil improves plant health (Gowariker *et al.* 2009, Lynch 1995). A three-dimensional (3D) deployment of roots in the soil to reach pockets of water and nutrients is called a root system architecture (RSA) (Lynch 1995). Likely, in crop production, RSA directly influences plant growth and yield depending on soil conditions. Under water-deficient conditions, for example, plants with deep-type RSA reach their roots into deeper soil regions containing adequate water and are able to produce more biomass (Uga *et al.* 2013). Each soil condition should be overcome with an ideal RSA. Improvement of the RSA according to the soil condition is a strategy to enhance plant productivity (Uga 2021, Uga *et al.* 2015). In crop breeding, RSA phenotyping by quantifying its components is essential for RSA improvement.

In monocotyledonous crops, RSA consists of the radicle, crown roots, and lateral roots. In dicotyledonous crops, RSA consists of the radicle plus lateral roots. The radicle and crown roots in monocotyledonous crops determine root distribution in soil. In dicotyledonous crops, the root distribution is determined by radicle and lateral roots. Lateral roots are also responsible for root density in soil. Measuring the placement of these roots in the soil is RSA phenotyping. Typical RSA phenotyping consists of two steps: sample preparation/collection and RSA quantification. In both steps, a conventional RSA phenotyping is labor intensive and throughput is very low because roots need to be dug out and washed before measurement (Böhm 1979). In recent years, digital phenotyping has automated the measurement process (Omari *et al.* 2020, Perez-Sanz *et al.* 2017, Walter *et al.* 2015). In this review, we introduce methods to improve the efficiency of crop RSA phenotyping through digitalization and its accompanying automation by comparison of analog and digital phenotyping.

## Digital phenotyping

Generally, the term “digital phenotyping” means “accelerated and automated phenotyping using informative digital data” (Debauche *et al.* 2017, Insel 2018, Ruckelshausen and Busemeyer 2015). In medical science, digital phenotyping emphasizes objective judgment that does not depend on human skills (Insel 2018), but in plant science, digital phenotyping emphasizes improving efficiency by automating labor-intensive tasks (Debauche *et al.* 2017, Ruckelshausen and Busemeyer 2015). In above-ground measurements, the term digital phenotyping has been used with informative digital data such as X-ray CT (computed tomography) images, hyperspectral images, and environmental data obtained from sensors. In general, phenotyping using small digital data such as digital photographs is not included in digital phenotyping. Due to the labor-intensive nature of root phenotyping, we have considered all phenotypic measurements for RSA from digital data as digital phenotyping in this study. Quantification methods that do not use digital data, therefore, are defined as “analog phenotyping”.

## Sample classification

**Fig. 1.**
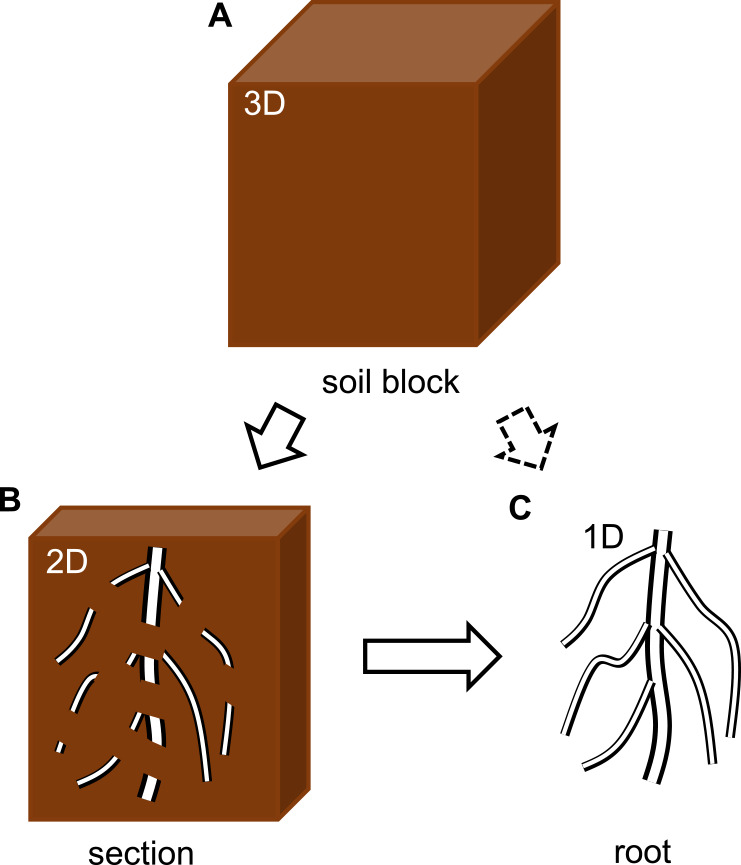
Three types of root samples. (A) block sample in which has 3D information of root distribution. (B) section sample which has 2D information of root distribution. The section sample is derived from the block sample. (C) section sample which has only 1D information. The root sample is derived from the section or block samples.

Whether plants are grown outdoors or indoors, there are three main types of samples for measuring roots, i.e., block, section, and root samples (Fig. 1). The block sample is a soil block including roots (Fig. 1A). The block size depends on the sampling method and is roughly up to 10000 cubic centimeters (Teramoto *et al.* 2019). It contains information on the 3D spatial distribution of roots, but because the soil is opaque, non-destructive measurement techniques are required. The section sample is a type of block sample that focuses on the visible roots exposed in a cross section (Fig. 1B), resulting in a two-dimensional (2D) spatial distribution of roots. The root sample is obtained by removing the soil from section and block samples (Fig. 1C). Because information of spatial root distribution in the soil is lost, only one-dimensional (1D) information will be obtained. With a little effort, it is also possible to obtain 2D or 3D data from root samples; 2D and 3D data could be indirectly obtained from divided section samples (Kitomi *et al.* 2020, Oyanagi *et al.* 1993, Uga *et al.* 2013) and divided block samples (Buczko *et al.* 2009, Kuchenbuch *et al.* 2009), respectively. The following three sections introduce how to prepare, digitize, and quantify block, section, and root samples.

## Block sample

### Sample preparation

Pot cultivation is an easy method for obtaining block samples; a block sample with the volume of the pot will be obtained. Pot size depends on the crop and growing period, but pots with a diameter of about 15–20 cm are usually used (Oyanagi *et al.* 1993, Teramoto *et al.* 2020). For non-destructive measurements, pots with a smaller diameter of under 10 cm are often used (Pflugfelder *et al.* 2017, Yoshida *et al.* 2020). When investigating the depth distribution of roots, thin tubes are used instead of pots (Iseki *et al.* 2018, Lafitte *et al.* 2001). Dividing the soil into smaller volumes allows estimation of root distribution in soil.

A round monolith is an iron or steel cylinder used to collect block samples containing a certain volume of root zone (Böhm 1979, Kang *et al.* 1994, Kano *et al.* 2011, Teramoto *et al.* 2019, Wade *et al.* 2015, Yoshino *et al.* 2019). Although hammering the round monolith into the ground is common, heavy machinery may be used to save labor (Teramoto *et al.* 2019). Because paddy field soil is sticky, sampled soil blocks do not easily collapse. Therefore, a round monolith is often used for rice plants. The sampled soil blocks could be divided to quantify root biomass at specific depths (Kang *et al.* 1994).

Core sampling is done by hammering thin vertical cylinders into the ground (Böhm 1979, Fehrenbacher and Alexander 1955, Yoshino *et al.* 2019). Due to the shape of the sampler, this method can only evaluate the 2D vertical root distribution. To examine the 3D root distribution, sampling at multiple locations is required (Böhm 1979, Fehrenbacher and Alexander 1955).

### Digitization and quantification

In analog phenotyping, root traits in the block sample cannot be evaluated because the roots are buried in the soil; soil blocks need to be cut open to make the section samples available. For rapid root counting, the roots are washed to make the root samples observable (Bennie *et al.* 1987). In digital phenotyping, the rapid root density estimation method was developed by counting roots of the section samples with a fluorescence imaging system (Wasson *et al.* 2016). An X-ray CT or magnetic resonance imaging (MRI) methodology is commonly used to directly quantify the roots in a block sample in a nondestructive manner. The resulting image is then analyzed using 3D phenotyping software (Atkinson *et al.* 2019, Pflugfelder *et al.* 2017, Teramoto *et al.* 2020, van Dusschoten *et al.* 2016). Each software has its own characteristics, and it is recommended to use them for different purposes.

Most software evaluates 3D root distribution in the soil by isolating root segments because root segmentation is easy to implement. For example, RootViz3D (Tracy *et al.* 2012), RooTrak (Mairhofer *et al.* 2012), and Root1 (Flavel *et al.* 2017) segment roots by employing root tracking algorithms. Rootine (Gao *et al.* 2019b, Phalempin *et al.* 2021) and RootForce (Gerth *et al.* 2021) recognize the tubular structure of roots. Tracking algorithms is a top-down approach in which segment root objects are based on a reference provided by previously studied data or human recognition, whereas a data-driven method such as segmentation recognizing tubular structure is a bottom-up approach (Mairhofer *et al.* 2012). Generally, the bottom-up approach is easy to automate because it requires no human intervention. The top-down approach is more difficult to automate, but by indicating root structure, complex root structures that cannot be addressed by the bottom-up approach can be accurately segmented.

**Fig. 2.**
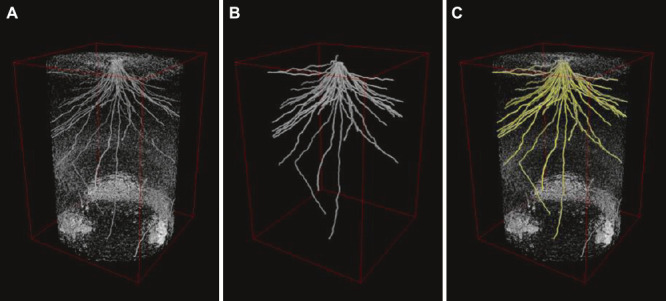
Three-dimensional rendering of RSA of a 42-day-old upland rice, Kinandang Patong. (A) A root-segmented image constructed by RSAvis3D. (B) A vector image constructed by RSAtrace3D. (C) The root-segmented image overlayed with the vector image colored yellow. The root system in a cylinder of 18 cm diameter and 25 cm height was visualized.

Recently, we developed a software, RSAvis3D, which uses a bottom-up approach to segment roots. We also created RSAtrace3D, which uses a top-down approach to vectorize roots. Combined, we are able to measure RSA traits (Teramoto *et al.* 2020, 2021). RSAvis3D is a software for visualizing RSA development of rice (Teramoto *et al.* 2020). In general, the diameter of the pot is smaller when segmenting the root system, including thin roots; 70 mm in Rootine and 56 mm in RootForce (Gao *et al.* 2019a, Gerth *et al.* 2021), but RSAvis3D extracts root segments from 200-mm-diameter pots by ignoring thin roots such as lateral roots (Teramoto *et al.* 2020), enabling scanning and visualization of a large RSA in a short period of time. To avoid human intervention, we adopted a bottom-up approach and used RSAvis3D to rapidly visualize the root system. To quantify RSA, the root system in RSAvis3D was vectorized with RSAtrace3D (Teramoto *et al.* 2021). Vectorization requires a top-down approach and human intervention, however, more detailed measurements of the root system can be made because the shape and connection of the root system can be expressed numerically. Extracted root segments by RSAvis3D, vectorized RSA image by RSAtrace3D, and extracted root segments overlayed with vectorized data are shown in Fig. 2A, 2B, and 2C, respectively. Using vector data, RSAtrace3D could measure not only root distribution in the soil but also rooting parameters such as rooting angle and length of individual root.

## Section sample

### Sample preparation

Trench profile method is a conventional method to observe spatial root distribution in the field soil (Böhm 1979, Teramoto and Uga 2020). In this method, a trench was dug up next to the plant to make a vertical section. Because trenching is labor intensive, heavy machinery is used. Roots exposed in the trench surface are to be quantified. In many cases, the trench surface is divided vertically or horizontally into subsections, and roots in each section are quantified to measure their distribution in the soil (Scarpare *et al.* 2019, Sekiya *et al.* 2013, Vansteenkiste *et al.* 2014).

Rhizotron is an artificially constructed soil environment to study plant roots. Root growth can be directly observed through the transparent sides. Because rhizotron was a large piece (several square meters) of equipment that involved digging trenches in the field and installing transparent sides, a smaller version that can be used in the laboratory was needed (Huck and Taylor 1982). Root box, which is several hundred to several thousand square centimeters of rhizotron, is widely used in the laboratory (Neufeld *et al.* 1989). Because of their convenience, root boxes have been used for large-scale, fully automated root system screening (Nagel *et al.* 2012).

The minirhizotron method allows researchers to observe roots in the soil by burying a scanner or digital camera (Cheng *et al.* 1991, Eshel and Beeckman 2013, Satomura *et al.* 2007). This method enables estimation of root growth dynamics including root turnover. As the data obtained is digital, only digital phenotyping is possible.

### Digitization and quantification

In analog phenotyping, root length on the section wall is mainly calculated by the line intersect method (Scarpare *et al.* 2019, Tennant 1975). In principle, given that a grid is overlayed on the section wall, the number of intersects of the grid with roots correlated with total root length on the section wall. However, this process is labor intensive and very low throughput.

In digital phenotyping, roots on the section surface are imaged by digital camera, and then those images are processed to quantify RSA traits (Joshi *et al.* 2017, Nagel *et al.* 2012, Shibusawa 1994, Teramoto and Uga 2020, Tognacchini *et al.* 2020). Root segments are isolated and skeletonized to calculate total root length on the section surface. The classical method of root segmentation is tracing the roots over the image, but this requires a great deal of effort (Teramoto and Uga 2020). Therefore, as in the case of block sample, top-down and bottom-up approaches are widely employed. For example, Pound and colleagues developed a segmentation software, RootNav, using a top-down approach, which utilizes a classification expectation–maximization algorithm to automatically determine root pixel connections (Pound *et al.* 2013). Narisetti and colleagues developed a segmentation software, saRIA, taking a bottom-up approach, which segments root regions by adaptive thresholding and morphological filtering (Narisetti *et al.* 2019).

**Fig. 3.**
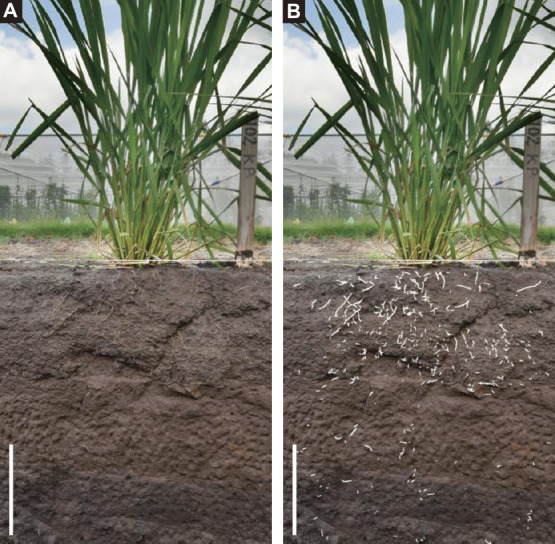
Trench profile image of a 113-day-old upland rice, Kinandang Patong. (A) A soil section image. (B) The soil section image overlayed with root-segmented image constructed by TrenchRoot-SEG. Root segments were highlighted in white. Bars indicate 20 cm.

A recent trend for analyzing section image data is semantic segmentation using convolutional neural networks (CNNs) (Jiang and Li 2020, Shen *et al.* 2020, Smith *et al.* 2020, Teramoto and Uga 2020, Wang *et al.* 2019, Yasrab *et al.* 2019). Because CNN is a deep neural network that can extract image features by introducing convolution layers, CNN is mostly applied to analyze image data (Gu *et al.* 2018). The CNN-based semantic segmentation consists of two major steps: model training and prediction with model. A CNN model is trained with labeled image data to learn the features of root segments, and root segments in images are semantically segmented using the trained CNN model. CNN-based semantic segmentation could separate the root segments in the image more efficiently than manual segmentation. In the case of trench profile images, it is estimated that CNN-based semantic segmentation including model training is over 100 times faster than manual segmentation (Teramoto and Uga 2020). An example of analysis of root segmentation from trench profile images using a CNN is shown in Fig. 3. The segmentation software developed recently are SegRoot (Wang *et al.* 2019), RootNav 2.0 (Yasrab *et al.* 2019), and TrenchRoot-SEG (Teramoto and Uga 2020).

## Root sample

### Sample preparation

Root samples are obtained by simply washing the roots out of the soil or by hydroponic culture (Takahashi and Pradal 2021). Because there is no soil or other support, the information obtained is 1D.

### Digitization and quantification

In analog phenotyping, the simplest phenotyping is measuring maximum root length by ruler (Kitomi *et al.* 2018, Obara *et al.* 2014), counting root number (Obara *et al.* 2014), and weighing root dry weight (Obara *et al.* 2014, Teramoto *et al.* 2019). Total root length is more difficult to measure, but as in section sample, can be estimated by the line intersect method (Tennant 1975). In digital phenotyping, the root sample is digitized by spreading roots out on a flat surface and imaging them by a scanner. Scanned images are analyzed by software specialized for root studies. The most popular software is WinRHIZO^TM^ (Regent Instrument, Canada). WinRHIZO uses a proprietary measurement algorithm to calculate distribution of root traits such as root length and diameter from scanned images. The number of parameters to be set by the user is small, and it is widely used in many RSA studies (Kashiwagi *et al.* 2005, Kawakatsu *et al.* 2021, McPhee 2005, Suematsu *et al.* 2017). Since WinRHIZO is a commercial product, the algorithm for the measurement is not public. To circumvent this potential complication, a number of open source software alternatives to WinRHIZO have been created (Pierret *et al.* 2013, Seethepalli *et al.* 2021, Tajima and Kato 2013).

## Bottlenecks for automated phenotyping

**Fig. 4.**
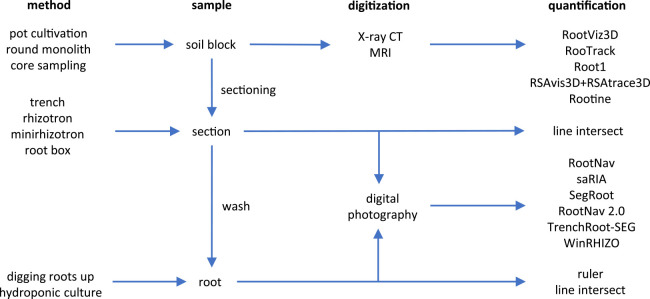
Relationships between sampling, digitizing, and quantifying methods.

We summarized the relationships between sampling, digitizing, and quantifying methods we introduced above (Fig. 4) and summarized the characteristics of each digital phenotyping method (Table 1). In the case of block sample, both top-down and bottom-up approaches are popular. Among them, some bottom-up approaches enable fully automated phenotyping (Gao *et al.* 2019b, Phalempin *et al.* 2021, Teramoto *et al.* 2020). Other software that is not fully automated is also used for different purposes, e.g., RSAtrace3D vectorizes RSA in X-ray CT images semi-automatically (Teramoto *et al.* 2021). This semi-automatic process is one bottleneck to full automation for analyzing block samples. As for section samples, fully automatic measurement techniques using CNNs have been reported in recent years, and it is believed that fully automatic measurement is becoming more and more popular (Joshi *et al.* 2017, Nagel *et al.* 2012, Shibusawa 1994, Teramoto and Uga 2020, Tognacchini *et al.* 2020). In the case of root sample, software using scanned images for root measurements such as WinRHIZO is fully automated. However, spreading roots for scanning is a labor-intensive task. For example, Kawakatsu and colleagues obtained scanned root images from 183 rice plants for WinRHIZO analysis (Kawakatsu *et al.* 2021). The total number of scanned images was about 2400, and it took six months for a laboratory assistant to acquire all these images. Therefore, unless the process of spreading roots is streamlined, root system phenotyping using scanned images will not be high-throughput.

## Conclusion and future perspective

In this review, we introduced digital phenotyping methods for RSA measurements, along with the characteristics of each method. Specifically, digital phenotyping with the section sample has been highly automated by employing CNNs (Table 1). The manual processes in digital phenotyping, like spreading roots before imaging, require improvement if high-throughput digitization is to be achieved. In above-ground measurements, techniques that can measure samples even when they overlap are becoming more popular. For example, prediction of branches hidden by leaves (Isokane *et al.* 2018) and measurements of seed shape of overlapping seeds (Toda *et al.* 2020) were developed with CNNs. It is predicted, therefore, that the technology to measure unseparated roots using CNNs will be developed in the future. In block samples, semantic segmentation has been fully automated by using CNNs, but vectorization of the root systems, which is needed to measure more complex traits, has been mostly semi-automatic (Teramoto *et al.* 2021). In medical science, fully automated vectorization algorism such as Segmentation-Less, Automated, Vascular Vectorization for the neurovascular network has been developed (Mihelic *et al.* 2021). If this algorithm could be applied to the vectorization of root systems, vectorization of the root system would become fully automated, which would accelerate RSA research.

Vector data can be used to quantify complex traits that cannot be calculated from image data. These data can be used to compare differences in root system traits between varieties, however, they are also useful root system model and simulation studies necessary to evaluate the performance of the root system under various environmental conditions (Lynch *et al.* 1997, Pagès *et al.* 1989, Postma *et al.* 2017, Takahashi and Pradal 2021). Thus, vector data have the potential to provide useful information for breeding crops that overcome growing climate issues. Vector data is, however, not yet widespread enough to enact the desired significant impact. Recent plant data deposit sites such as Quantitative Plant (https://www.quantitative-plant.org) and PlantCV (Fahlgren *et al.* 2015) mainly collect image data. Vector data remains a minority in such databases. One major reason for the lack of vector data is that conversion from image to vector is labor intensive. When digital phenotyping and vector data acquisition methodologies improve, we expect root system phenotyping to be widely incorporated into the breeding process.

## Author Contribution Statement

S.T. wrote the manuscript. Y.U. revised the manuscript.

## Figures and Tables

**Table 1.** Characteristics of digital phenotyping method for block, section, and root samples

Sample type	Digitization tool	Major analysis method	Other required effort	Automation
block	X-ray CT or MRI	any	–	yes/no
section	digital camera	CNN	–	yes
root	scanner	WinRHIZO	spreading roots	yes/no
